# Periodontitis and Subsequent Risk of Cataract: Results From Real-World Practice

**DOI:** 10.3389/fmed.2022.721119

**Published:** 2022-02-04

**Authors:** Li-Jen Yeh, Te-Chun Shen, Kuo-Ting Sun, Cheng-Li Lin, Ning-Yi Hsia

**Affiliations:** ^1^Department of Craniofacial Orthodontics, Chang Gung Memorial Hospital, Taoyuan, Taiwan; ^2^Graduate Institute of Craniofacial and Dental Science, Chang Gung University, Taoyuan, Taiwan; ^3^Department of Internal Medicine, China Medical University Hospital, Taichung, Taiwan; ^4^School of Medicine, China Medical University, Taichung, Taiwan; ^5^Department of Pediatric Dentistry, China Medical University Hospital, Taichung, Taiwan; ^6^Management Office for Health Data, China Medical University Hospital, Taichung, Taiwan; ^7^Department of Ophthalmology, China Medical University Hospital, Taichung, Taiwan

**Keywords:** periodontitis, cataract, retrospective cohort study, epidemiology, risk factor

## Abstract

**Background:**

Periodontitis can lead to systemic inflammation and oxidative stress, contributing to the development of various diseases. Periodontitis could also be associated with several ocular diseases.

**Methods:**

We conducted a retrospective population-based cohort study using the National Health Insurance Research Database of Taiwan to evaluate the risk of cataract in people with and without periodontitis. We established a periodontitis cohort and a non-periodontitis cohort, which included 359,254 individuals between 2000 and 2012. Age, gender, and enrolled year were matched. All participants were monitored until the end of 2013. Cox proportional hazard models were applied to estimate hazard ratios (HRs) and confidence intervals (CIs).

**Results:**

Patients with periodontitis had a significantly higher risk to develop cataract than those without periodontitis [10.7 vs. 7.91 per 1,000 person-years, crude HR = 1.35 (95% CI = 1.32–1.39), and adjusted HR = 1.33 (95% CI = 1.30–1.36)]. The significant levels remained the same after stratifying by age, gender, presence of comorbidity, and use of corticosteroid. In addition, we found that diabetes mellitus and hyperlipidemia had a synergistic effect in the interaction of periodontitis and cataract development.

**Conclusion:**

Patients with periodontitis have a higher risk of cataract development than those without periodontitis. Such patients may request frequent ocular health check-up. Further studies should be performed to confirm the association and to understand the mechanisms.

## Introduction

Periodontitis is a common disorder that could damage the bone and tissue supporting the tooth (1). It could also induce chronic infection, systemic inflammation, and oxidative stress (2). Poor periodontal status significantly reduces life quality and general health (3). A link between periodontitis and various diseases, including cardiovascular disease (4), diabetes mellitus (5), metabolic syndrome (6), osteoporosis (7), gastrointestinal disease (8), respiratory disease (9), and autoimmune disease, is suggested (10). In addition, periodontitis can affect the development of several ocular diseases (11–13).

Cataract is caused by a build-up of protein that clouds the lens, which can lead to blurred vision and blindness (14). Around 95 million people worldwide are affected by cataract, which remains the leading cause of blindness in middle- and low-income countries (15). Many causative factors could promote the development of cataract, which include old age, female gender, smoking, sunlight exposure, family history, diabetes mellitus, cardiovascular disease, chronic airway disease, corticosteroid use, and ocular infection or inflammation (16, 17).

The association between periodontitis and cataract remains largely unknown. As we know, periodontitis may increase the systemic inflammatory reaction, and cataract could be initiated and exacerbated by the result of chronic inflammation (18–20). Furthermore, periodontitis-induced oxidative stress may also play a crucial role in the development of cataract (21–23). In a cross-sectional study, Gervasio et al. (24) examined many institutionalized geriatric residents and reported that the prevalence of periodontitis and cataract were both predominant. However, the exact relationship between these two common diseases is not well-established to date. Therefore, we aimed to conduct a retrospective population-based cohort study based on the National Health Insurance Research Database (NHIRD) in Taiwan to evaluate the association of periodontitis and subsequent development of cataract.

## Materials and Methods

### Data Source

The National Health Insurance (NHI) program operated since 1995, with more than 99.9% of Taiwan citizens enrolled. The NHIRD is managed and updated by the National Health Research Institutes between 1995 and 2013. We applied the Longitudinal Health Insurance Database 2000 (LHID2000), a subset of NHIRD, to complete the study. The database included detailed medical information of 1,000,000 people randomly selected in 2000, such as demographic status, diagnostic code, medication, and procedure claims was available. The study was approved by the Research Ethics Committee of the China Medical University and Hospital (CMUH-104-REC2-115). Informed consent was unnecessary for the de-identified data and waived by the Research Ethics Committee.

### Study Population

We selected newly diagnosed adult patients with periodontitis [International Classification of Diseases, 9th Revision, Clinical Modification (ICD-9-CM) codes 523.3 and 523.4] between 2000 and 2012 as the periodontitis group (*n* = 179,627). The date of diagnosis was defined as the index date. We excluded those with incomplete demographic data and those with cataract before the index date. Thus, we selected the same number of adult individuals without periodontitis as the comparison group. Age, gender, and index year were matched between the periodontitis and the non-periodontitis groups. The exclusion criteria were the same as the periodontitis group. All participants were monitored until (1) the development of cataract, (2) withdrawal from the NHI program, (3) death, or (4) the end of 2013.

### Study Outcome and Confounders

The primary outcome of the study was the diagnosis of cataract (ICD-9-CM code 366). We further identified several comorbidities that may be potential risk factors for cataract and the most related medication, corticosteroid, as confounders. Detailed comorbidities included hypertension (ICD-9-CM codes 401–405), diabetes mellitus (ICD-9-CM code 250), hyperlipidemia (ICD-9-CM code 272), asthma/chronic obstructive pulmonary disease (COPD) (ICD-9-CM codes 493 and 496), chronic liver disease and cirrhosis (CLD; ICD-9-CM code 571), chronic kidney disease (CKD; ICD-9-CM code 585), and rheumatic diseases (ICD-9-CM codes 446.5, 710.0–710.4, 714.0–714.2, 714.8, and 725).

### Statistical Analysis

We applied chi-squared test and *t*-test to compare the distribution of baseline characteristics for categorical and continuous variables. We have drawn the Kaplan-Meier curves followed by testing inter-group differences with a log-rank test to evaluate the cumulative incidence of cataract in both groups. Cox proportional hazard models were used to estimate the hazard ratios (HRs) and 95% confidence intervals (CIs). Multivariate Cox models were used to estimate the adjusted HRs (aHRs) and 95% CIs after controlling age, sex, comorbidities, and corticosteroid use, which were significant in the univariate model. All the analyses were performed using STATA statistical software (StataCorp. 2015, R 14, StataCorp LP). The level of significance was set at 0.05 using a two-tailed test.

## Results

**Figure 1:**
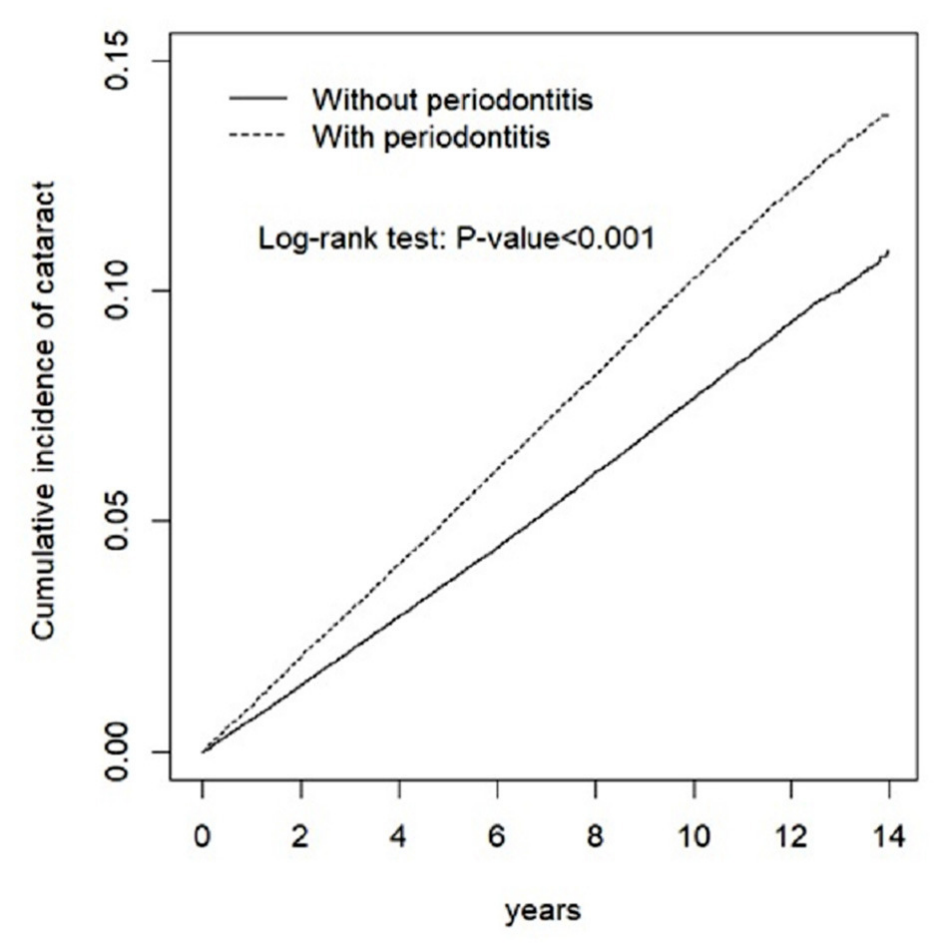
Cumulative incidence of cataract for individuals with and without periodontitis.

This study included 179,627 periodontitis patients and 179,627 non-periodontitis individuals that displayed similar distributions of age and gender (Table 1). The mean age of the periodontitis group was 40.3 (standard deviation = 13.5) years, 51.0% of whom were women. The prevalence rates of hypertension, diabetes mellitus, hyperlipidemia, asthma/COPD, CLD, rheumatic diseases, and corticosteroid use were all greater in patients with periodontitis than those without periodontitis (*p* < 0.001). Figure 1 shows that the cumulative incidence of cataract was higher in the periodontitis group than in the non-periodontitis group (*p* < 0.001) after a 14-year follow-up.

**Table 1:** Baseline characteristics for individuals with and without periodontitis.

	**Periodontitis**				
	**No *N* = 179,627**		**Yes *N* = 179,627**		
	***n***	**%**	***n***	**%**	***p* ^#^**
**Age**					>0.99
20–49	134,973	75.1	134,973	75.1	
50–64	36,555	20.4	36,555	20.4	
≥ 65	8,099	4.51	8,099	4.51	
Mean ±SD	40.0	±13.9	40.3	±13.5	0.001
**Gender**					>0.99
Women	91,658	51.0	91,658	51.0	
Men	87,969	49.0	87,969	49.0	
**Comorbidity**					
Hypertension	24,924	13.9	27,476	15.3	<0.001
Diabetes mellitus	4,278	2.38	4,611	2.57	<0.001
Hyperlipidemia	18,266	10.2	24,949	13.9	<0.001
Asthma/COPD	15,238	8.48	18,135	10.1	<0.001
CLD	25,978	14.5	34,272	19.1	<0.001
CKD	1,048	0.58	1,102	0.61	0.39
Rheumatic diseases	2,627	1.46	3,715	2.07	<0.001
**Medication**					
Corticosteroid use	4,941	2.75	5,668	3.16	<0.001

*CKD, chronic kidney disease; CLD, chronic liver disease and cirrhosis; COPD, chronic obstructive pulmonary disease; SD, standard deviation*.

# *Chi-squired test and t-test*.

The incidence density of cataract was greater in the periodontitis group than in the non-periodontitis group (10.7 vs. 7.91 per 1,000 person-years) (Table 2). The multivariable Cox model estimated aHR of cataract was 1.33 (95% CI = 1.30–1.36) for the periodontitis group compared with the non-periodontitis group after controlling for age, gender, comorbidities, and corticosteroid use. Compared with those <50 years of age, the aHRs were 10.5 in the 50–64 years age group (95% CI = 10.1–10.8) and 19.8 in the ≥65 years age group (95% CI = 19.1–20.7). Compared with men, women had an aHR of 1.33 (95% CI = 1.30–1.36) for cataract development. Compared with non-corticosteroid users, corticosteroid users had an aHR of 1.21 (95% CI = 1.15–1.27) for cataract development. Compared with non-diabetes mellitus individuals, diabetes mellitus patients had an aHR of 1.90 (95% CI = 1.82–1.98) for cataract development. Furthermore, aHRs were 1.39 (95% CI = 1.35–1.43) for individuals with hypertension, 1.28 (95% CI = 1.25–1.32) for individuals with hyperlipidemia, 1.25 (95% CI = 1.14–1.37) for individuals with CKD, 1.24 (95% CI = 1.20–1.27) for individuals with CLD, 1.21 (95% CI = 1.13–1.29) for individuals with rheumatic diseases, and 1.19 (95% CI = 1.15–1.23) for individuals with asthma/COPD.

**Table 2:** Associated factor analysis for cataract.

	**Event**	**PY**	**Rate^#^**	**Crude HR (95% CI)**	**Adjusted HR^†^ (95% CI)**
**Periodontitis**					
No	10,937	1383000	7.91	1.00	1.00
Yes	15.078	1407568	10.7	1.35 (1.32–1.39)^***^	1.33 (1.30–1.36)^***^
**Age**					
20–49	5,394	2220424	2.43	1.00	1.00
50–64	15,098	485717	31.1	13.3 (12.9–13.7)^***^	10.5 (10.1–10.8)^***^
≥ 65	5,523	84427	65.4	28.7 (27.7–29.8)^***^	19.8 (19.1–20.7)^***^
**Gender**					
Women	14,487	1431226	10.1	1.19 (1.16–1.22)^***^	1.33 (1.30–1.36)^***^
Men	11,528	1359343	8.48	1.00	1.00
**Comorbidity**					
**Hypertension**					
No	14,757	2452127	6.02	1.00	1.00
Yes	11,258	338441	33.3	5.62 (5.49–5.76)^***^	1.39 (1.35–1.43)^***^
**Diabetes mellitus**					
No	23,094	2741295	8.42	1.00	1.00
Yes	2,921	49273	59.3	7.17 (6.90–7.46)^***^	1.90 (1.82–1.98)^***^
**Hyperlipidemia**					
No	17,709	2508842	7.06	1.00	1.00
Yes	8,306	281726	29.5	4.24 (4.13–4.35)^***^	1.28 (1.25–1.32)^***^
**Asthma/COPD**					
No	21,094	2577384	8.18	1.00	1.00
Yes	4,921	213185	23.1	2.86 (2.77–2.95)^***^	1.19 (1.15–1.23)^***^
**CLD**					
No	18,412	2353531	7.82	1.00	1.00
Yes	7,603	437037	17.4	2.23 (2.17–2.29)^***^	1.24 (1.20–1.27)^***^
**CKD**					
No	25,546	2779068	9.19	1.00	1.00
Yes	469	11500	40.8	4.50 (4.11–4.93)^***^	1.25 (1.14–1.37)^***^
**Rheumatic diseases**					
No	25,067	2750306	9.11	1.00	1.00
Yes	948	40263	23.6	2.61 (2.45–2.78)^***^	1.21 (1.13–1.29)^***^
**Medication**					
Corticosteroid use					
No	24,368	2731200	8.92	1.00	1.00
Yes	1,647	59368	27.7	3.16 (3.01–3.33)^***^	1.21 (1.15–1.27)^***^

*CI, confidence interval; CKD, chronic kidney disease; CLD, chronic liver disease and cirrhosis; COPD, chronic obstructive pulmonary disease; HR, hazard ratio; PY, person-years*.

# *Incidence rate per 1,000 person-years*.

† *Multivariable analysis including age, gender, comorbidities, and corticosteroid use*.

*** *p < 0.001*.

Table 3 shows incidences and HRs of cataract for both study groups, stratified by age, gender, the presence of comorbidity, and corticosteroid use. The incidences of cataract were higher in elders, women, those with comorbidity, and corticosteroid users between both groups. The periodontitis to non-periodontitis group aHRs were significant for the young age group (1.27, 95% CI = 1.20–1.34), middle age group (1.28, 95% CI = 1.24–1.32), and old age group (1.54, 95% CI = 1.45–1.62). The periodontitis to non-periodontitis group aHRs were significant for women (1.28, 95% CI = 1.24–1.33) and men (1.39, 95% CI = 1.34–1.44). The periodontitis to non-periodontitis group aHRs were significant for those without comorbidity (1.44, 95% CI = 1.38–1.51) and those with comorbidity (1.30, 95% CI = 1.26–1.34). The periodontitis to non-periodontitis group aHRs were significant for non-corticosteroid users (1.33, 95% CI = 1.29–1.36) and corticosteroid users (1.34, 95% CI = 1.22–1.48).

**Table 3:** Incidences and hazard ratios of cataract for individuals with and without periodontitis by age, gender, comorbidity, and corticosteroid use.

	**Periodontitis**							
	**No**			**Yes**				
	**Event**	**PY**	**Rate^#^**	**Event**	**PY**	**Rate^#^**	**Crude HR (95% CI)**	**Adjusted HR^†^ (95% CI)**
**Age**								
20–49	2,253	1096510	2.05	3,141	1123914	2.79	1.35 (1.28–1.43)^***^	1.27 (1.20–1.34)^***^
50–64	6,512	243514	26.7	8,586	242203	35.5	1.33 (1.29–1.37)^***^	1.28 (1.24–1.32)^***^
≥ 65	2,172	42976	50.5	3,351	41451	80.8	1.59 (1.51–1.68)^***^	1.54 (1.45–1.62)^***^
**Gender**								
Women	6,280	711630	8.82	8,207	719596	11.4	1.29 (1.25–1.34)^***^	1.28 (1.24–1.33)^***^
Men	4,657	671370	6.94	6,871	687972	9.99	1.44 (1.39–1.49)^***^	1.39 (1.34–1.44)^***^
**Comorbidity^‡^**								
No	3,845	998982	3.85	4,496	928178	4.84	1.25 (1.20–1.31)^***^	1.44 (1.38–1.51)^***^
Yes	7,092	384018	18.5	10,582	479390	22.1	1.20 (1.16–1.23)^***^	1.30 (1.26–1.34)^***^
**Corticosteroid**								
No	10,289	1355838	7.59	14,079	1375362	10.2	1.35 (1.31–1.38)^***^	1.33 (1.29–1.36)^***^
Yes	648	27162	23.9	999	32206	31.0	1.30 (1.18–1.44)^***^	1.34 (1.22–1.48)^***^

*CI, confidence interval; HR, hazard ratio; PY, person-years*.

# *Incidence rate per 1,000 person-years*.

† *Multivariable analysis including age, gender, comorbidities, and corticosteroid use*.

‡ *Individuals with any comorbidity of hypertension, diabetes mellitus, hyperlipidemia, asthma/COPD, CLD, CKD, and rheumatic disease were classified into the comorbidity group*.

*** *p < 0.001*.

Table 4 presents the risk of cataract associated with interactions between periodontitis and the comorbidity of hypertension, diabetes mellitus, and hyperlipidemia. Those with both periodontitis and diabetes mellitus (aHR = 2.66, 95% CI = 2.52–2.81, interaction *p* = 0.007) and those with both periodontitis and hyperlipidemia (aHR = 2.00, 95% CI = 1.93–2.08, interaction *p* = 0.002) presented a significantly higher risk. Moreover, patients with more comorbidities of hypertension, diabetes mellitus, and hyperlipidemia had a trend to have a higher risk of cataract (interaction *p* = 0.08).

**Table 4:** Cox proportional hazards regression analysis for the risk of cataract-associated periodontitis and hypertension, diabetes mellitus, and hyperlipidemia.

**Variables**		**Total *N***	**Cataract *N***	**Adjusted HR^†^ (95% CI)**	***p* ^#^**
Periodontitis	Hypertension				0.16
No	No	154,703	6,316	1 (Reference)	
No	Yes	24,924	4,621	1.40 (1.35–1.46)^***^	
Yes	No	152,151	8,441	1.38 (1.33–1.42)^***^	
Yes	Yes	27,476	6,637	1.84 (1.77–1.91)^***^	
Periodontitis	Diabetes mellitus				0.007
No	No	175,349	9,715	1 (Reference)	
No	Yes	4,278	1,222	2.08 (1.96–2.21)^***^	
Yes	No	175,016	13,379	1.37 (1.33–1.40)^***^	
Yes	Yes	4,611	1,699	2.66 (2.52–2.81)^***^	
Periodontitis	Hyperlipidemia				0.002
No	No	161,361	7,818	1 (Reference)	
No	Yes	18,266	3,119	1.63 (1.56–1.70)^***^	
Yes	No	154,678	9,891	1.37 (1.33–1.41)^***^	
Yes	Yes	24,949	5,187	2.00 (1.93–2.08)^***^	
Periodontitis	Triple H				0.08
No	0	145,595	5,090	1 (Reference)	
No	1	22,513	3,301	1.55 (1.48–1.62)^***^	
No	2	9,602	1,977	1.93 (1.82–2.03)^***^	
No	3	1,917	569	2.65 (2.43–2.90)^***^	
Yes	0	139,156	6,432	1.38 (1.33–1.43)^***^	
Yes	1	26,020	4,627	2.03 (1.95–2.12)^***^	
Yes	2	12,337	3,161	2.36 (2.25–2.47)^***^	
Yes	3	2,114	858	3.58 (3.32–3.86)^***^	

*CI, confidence interval; HR, hazard ratio*.

† *Model was adjusted for age, sex, comorbidities, and corticosteroid use*.

# *p-value for interaction*.

*** *p < 0.001*.

## Discussion

This retrospective population-based cohort study analyzed the incidence of cataract in individuals with and without periodontitis. Results showed that periodontitis patients were associated with a higher risk of cataract development than non-periodontitis individuals. As expected, the incidences of cataract were higher in older people than in younger people, in women than in men, in those with comorbidity than in those without comorbidity, and in corticosteroid users than in non-corticosteroid users. Furthermore, cataract risk was significantly higher in the periodontitis group than in the comparison group even after stratifying by age, gender, the presence of comorbidity, or corticosteroid use. Moreover, we found that diabetes mellitus and hyperlipidemia had a synergistic effect in the interaction of periodontitis and cataract development.

In the present study, we have evaluated several potential risk factors and their impacts on cataract development. Overall, diabetes mellitus played the most important role in the development of cataract, followed by hypertension, hyperlipidemia, CKD, CLD, corticosteroid use, rheumatic diseases, and asthma/COPD. Triple H (hypertension, hyperglycemia, and hyperlipidemia), metabolic syndrome, atherosclerosis, and cardiovascular diseases would have the most impact to cataract development; these findings correlated with previous studies (14, 15). However, the association between chronic liver disease and cataract or rheumatic diseases and cataract needs further investigations.

The potential mechanisms of the association between periodontitis and cataract remained unclear, but several hypotheses have been suggested. First, odontogenic ocular infections may directly influence the development of cataract. Both mouth and teeth are known reservoirs for many pathogens; therefore, periodontitis may contribute to repeated or chronic ocular infections (25). In a large-scale cohort study, Chau et al. (11). have reported that patients with periodontal disease (*n* = 467,170) are at a higher risk of infectious scleritis (aHR = 1.270, 95% CI = 1.114–1.449), uveitis (aHR = 1.144, 95% CI = 1.074–1.218), and infectious keratitis (aHR = 1.094, 95% CI = 1.030–1.161) than those without periodontal disease (*n* = 467,170). These infectious conditions have shown to be risk factors of cataract (15). Second, oral microbiome from periodontitis can cause immune responses to exacerbate cataractogenesis. Some observations implied periodontitis-induced systemic inflammation and oxidative stress in the pathogenesis of eye diseases (15, 26, 27). That is, periodontal microbiota may trigger immune dysfunction in the oro-optic-network and promote the development of cataract. Third, the impact of periodontitis in the induction and progression of ocular diseases such as diabetic retinopathy, glaucoma, and age-related macular degeneration has been identified (12, 13, 27, 28). The pathophysiology between periodontitis and these ocular complications may be similar to that of cataract. Finally, smoking, lower socioeconomic status, and shared comorbidities, such as diabetes mellitus, hypertension, hyperlipidemia, cardiovascular disease, and chronic airway disease, may also contribute to the development of cataract in periodontitis patients.

The primary strength of the study is the use of population-based data that are highly representative of the general population. No difference was found in the demographic distribution between LHID2000 and the original NHIRD. In addition, the universal coverage in the insurance system ensures that all citizens can have no access barriers to health care (29). Moreover, the NHIRD reflected a real-world scenario and the results of clinical practices.

Certain limitations should be considered in the study. First, the diagnosis is only based on ICD code, but the NHIRD has been validated and the results showed the data was reliable (30). Second, the NHIRD does not contain detailed information on smoking habits, occupational or environmental exposure, body mass index, and family history, which may be confounding factors. Third, the database did not contain clinical variables such as dental and ocular findings, disease severity and subtype, laboratory data, culture reports, and imaging findings. Fourth, the treatment effects of periodontitis could not be well-evaluated in the database. Fifth, the follow-up period may be short for cataract development. Finally, the study could be biased because of possible unmeasured or unknown confounding variables.

## Conclusion

Patients with periodontitis are at a higher risk of cataract development than those without periodontitis. Such patients may request frequent ocular health check-up. Further studies should be performed to confirm the association and to understand the mechanisms.

## Data Availability Statement

The original contributions presented in the study are included in the article/supplementary material, further inquiries can be directed to the corresponding authors.

## Ethics Statement

The studies involving human participants were reviewed and approved by CMUH-104-REC2-115. Written informed consent for participation was not required for this study in accordance with the national legislation and the institutional requirements.

## Author Contributions

L-JY, T-CS, K-TS, and N-YH: study concept and design. L-JY, T-CS, K-TS, C-LL, and N-YH: acquisition of data. L-JY, T-CS, C-LL, and N-YH: data analysis. L-JY, T-CS, C-LL, and N-YH: writing. All authors contributed to the article and approved the submitted version.

## Funding

This study was supported in part by Taiwan Ministry of Health and Welfare Clinical Trial Center (MOHW109-TDU-B-212-114004) and China Medical University Hospital (DMR-110-033).

## Conflict of Interest

The authors declare that the research was conducted in the absence of any commercial or financial relationships that could be construed as a potential conflict of interest.

## Publisher's Note

All claims expressed in this article are solely those of the authors and do not necessarily represent those of their affiliated organizations, or those of the publisher, the editors and the reviewers. Any product that may be evaluated in this article, or claim that may be made by its manufacturer, is not guaranteed or endorsed by the publisher.
